# Fatal Case of Probable Invasive Aspergillosis after Five Years of Heart Transplant: A Case Report and Review of the Literature

**DOI:** 10.1155/2015/864545

**Published:** 2015-08-24

**Authors:** Toufik Mahfood Haddad, Mahesh Anantha Narayanan, Krista E. Shaw, Renuga Vivekanandan

**Affiliations:** ^1^Department of Internal Medicine, CHI Health Creighton University School of Medicine, 601 North 30th Street No. 5850, Omaha, NE 68131, USA; ^2^Medical School, Creighton University School of Medicine, Omaha, NE, USA; ^3^Division of Infectious Diseases, CHI Health Creighton University School of Medicine, Omaha, NE, USA

## Abstract

Invasive fungal infections are very common in solid organ transplants and occur most frequently in the first three months after transplant. A 49-year-old female with a history of two remote heart transplants with the most recent one occurring 5 years ago was admitted for increasing shortness of breath, cough, and fever. Computerized tomography (CT) scan of the chest showed left lower lung ground-glass and tree-in-bud opacities. She was started on broad spectrum antibiotics along with ganciclovir and micafungin. Ganciclovir was added due to the patient's past history of CMV infection and empiric fungal coverage with micafungin. Bronchoalveolar lavage (BAL) was performed as her respiratory status worsened and voriconazole was added for possible aspergillosis in combination therapy with micafungin. BAL galactomannan returned positive which was suggestive of aspergillosis. Patient worsened clinically and subsequently succumbed to cardiorespiratory arrest despite our best efforts. It is important to have a high degree of clinical suspicion for invasive aspergillosis in transplant patients even many years after transplant and initiate aggressive therapy due to poor outcomes.

## 1. Background

Heart transplant recipients are at risk of invasive fungal infections, a fatal complication of any transplant [1]. Neutropenia, graft versus host disease, corticosteroid therapy, and cytomegalovirus infection increase the risk of having invasive aspergillosis [2, 3]. With new changes in the immunosuppressive and antimicrobial prophylactic regimen over the past 30 years, the incidence of invasive aspergillosis has significantly decreased [4]. We hereby present a patient with a history of remote heart transplant and a retransplant 5 years ago due to CMV induced rejection that now presented with invasive aspergillosis leading to death in spite of being on multiple antifungal therapy.

## 2. Case Presentation

A 49-year-old Caucasian female presented to the emergency department (ED) with increasing shortness of breath and a productive cough of one-week duration along with associated fever and chills that began the day before she was hospitalized. The patient denied any hemoptysis, myalgia, or arthralgia as well as chest pain, orthopnea, and palpitations. The patient had undergone two cardiac transplants. The first was 15 years ago secondary to giant cell myocarditis and cardiogenic shock. The second retransplant occurred five years ago due to right sided heart failure and cytomegalovirus (CMV) induced chronic rejection. Four months ago, the patient experienced acute rejection grade IIR diagnosed by endocardial biopsy, which had improved with pulse-dose steroids for three days. A repeat biopsy a few days before this admission showed decreased inflammation with mild chronic interstitial infiltrate with no inclusion bodies or myocyte necrosis without evidence of graft vasculopathy. Her medications included mycophenolate mofetil and tacrolimus, along with tapering oral steroids.

Vital signs showed blood pressure of 107/70 mmHg, temperature of 100.4 F, heart rate of 103 bpm, respiratory rate of 18, and saturation of 93% on 4 liters of oxygen. The pharyngeal exam revealed erythema with transudative pharyngitis. On auscultation, she had coarse bilateral crackles without wheezes; heart sounds were regular without extra sounds, with a 2/6 systolic holosystolic murmur on the left lower sternal border (LLSB) and bilateral lower extremities pitting edema.

**Figure 1:**
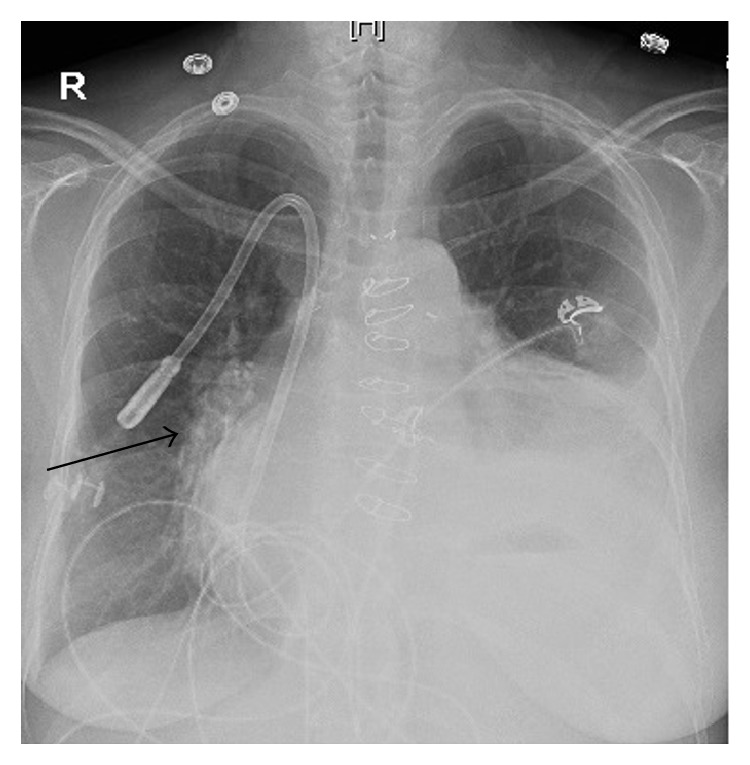
CXR showing airspace opacities with right hilar atelectasis and cardiomegaly.

**Figure 2:**
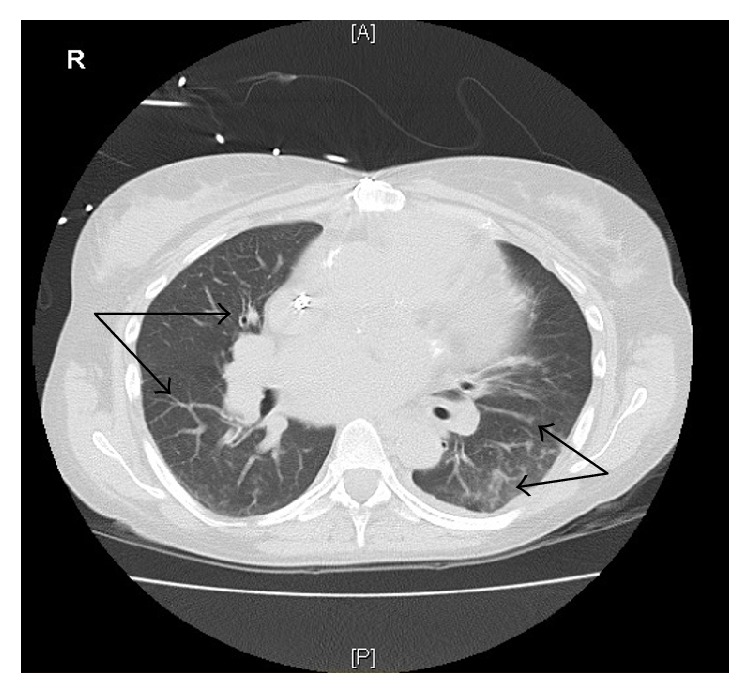
CT scan of the chest showing bilateral lungs ground-glass and tree-in-bud opacities.

Significant laboratory parameters included leukocytosis of 13,400 cells/mm^3^ with neutrophilic predominance, elevated creatinine correlated with end-stage renal disease, and lactic acidosis. Hepatic and coagulation parameters were within normal limits. Additionally, there were elevated cardiac biomarkers (troponin-I of 0.98 ng/mL without a rise or fall, along with normal CK MB at 3.6 ng/mL, and brain-natriuretic peptide of 25740 ng/L). A chest X-ray was performed and showed bilateral airspace opacities with right hilar atelectasis and cardiomegaly (Figure 1). A follow-up CT scan of the chest showed bilateral lungs ground-glass and tree-in-bud opacities (Figure 2) representing an infectious etiology.

The patient was started on broad spectrum antibiotics including vancomycin, piperacillin-tazobactam, and levofloxacin, in addition to ganciclovir and micafungin for empirical bacterial, viral, and fungal coverage. Micafungin was started for empirical fungal coverage and ganciclovir was added due to the patient's past history of CMV infection. Patient was diagnosed with possible invasive fungal infection with the presence of risk factors and having clinical criteria. Host risk factors included chronic immunosuppression with tacrolimus, CellCept, and corticosteroid. The clinical criteria were lower respiratory tract fungal disease with dense, well-circumscribed lesions.

An echocardiogram showed an ejection fraction of 15%, which was unchanged from previous studies. Initial blood aerobic, anaerobic, and fungal cultures revealed no growth. On day three of admission, the patient's AST and ALT were elevated to 3201 and 1242 units/L, respectively. Her INR was also elevated. Shortly thereafter, she became altered; her oxygen requirements increased and she went into multiorgan failure. Voriconazole was added for double coverage of possible invasive aspergillosis (IA), but the patient's clinical status did not improve. On day five, the patient underwent a bronchoscopy with bronchoalveolar lavage (BAL) which revealed a negative workup for herpes simplex virus, cytomegalovirus, legionella, mycoplasma, influenza, beta-D-glucan, and cryptococcal pathogens. However, the workup was positive for aspergillus galactomannan of 1.69 indexes, but it was negative in serum with 0.06 indexes (negative if less than 0.5). This probable diagnosis did not change the treatment plan, as the patient was already on optimal antifungal management with double antifungal treatment. The patient's status continued to deteriorate despite maximal respiratory support and maximized pressures. She worsened clinically and subsequently succumbed to cardiorespiratory arrest despite our best efforts.

## 3. Discussion

Invasive fungal infections (IFIs) are a major cause of morbidity and mortality in posttransplant patients. Multicenter surveillance data identified its occurrence to be 19% among organ transplant recipients with candidiasis being the most common one and aspergillosis being the second most common one [6]. The chance of fungal infection after heart transplant (HT) has decreased to 0.08 [7] with aspergillosis accounting for only 2.3–3.5% of post-HT infections [5, 8], which was decreased from 6.5% before 2000 [8].

Invasive aspergillosis (IA) is a severe, rapidly progressive, frequently fatal infection that occurs in immunocompromised patients and spreads beyond the respiratory tract to multiple different organs. Conversely, chronic aspergillosis infections, which usually occur in immunocompetent patients, progress over months to years and require prolonged antifungal treatment [9].

Patients at risk for IA are those with severe and prolonged neutropenia, advanced acquired immunodeficiency syndrome, or chronic granulomatous disease, and patients who have undergone posttransplantation, those using glucocorticoids, or those undergoing chemotherapy are also at risk for developing IA [2, 3].

The median time of onset of IA following transplantation depends on the type of transplant with most cases occurring within the first 3 months [10]. Half of the HT patients experienced an infection in the first year and 75% of these infections occurred within the first three months [5] (Table 1). A recent study showed that the chance of having aspergillosis after HT ranges between 46 and 52 days, with most of the infections occurring in the first 2-3 months [4, 11, 12]. Late aspergillosis cases (>3 months) after HT had higher rate for mortality and dissemination [8], with the rate of incidence being decreased significantly from >0.15 episodes/patient-month in the first 3 months to <0.05 episodes/patient-month after six months [12]. This demonstrates the uniqueness of this particular case as the patient acquired IA 5 years after transplant. While this is unusual, it solidifies the importance of remaining vigilant in regard to IA in all posttransplant patients regardless of the time elapsed since the transplant occurred.

The prophylactic use of antifungal agents is associated with actual and potential problems. Although several antifungal agents are available, the choice is not straightforward as each one has differing spectra of activity, pharmacological properties, toxicities, and costs [13]. The current data supports the use of prophylaxis for aspergillus in liver and lung transplant recipients and for* Candida* in liver, bowel, and pancreas transplant recipients [14]. In cardiac transplant recipients, neither ketoconazole nor clotrimazole significantly reduced invasive fungal infections per a meta-analysis conducted in 2004 [13]. However, recent studies showed benefits for providing a median of 20 days of targeted prophylaxis to post-heart transplant patients who exhibited risk factors for developing fungal infections [15].

IFIs are often diagnosed late because of their nonspecific clinical features and the poor sensitivity and specificity of currently available diagnostic tests. There are different classifications for IFI defined as possible, probable, or proven disease [16]. In our case, the patient was diagnosed for possible invasive fungal infection initially with host risk factors and presence of clinical criteria. She was treated empirically until her BAL galactomannan returned positive which made the diagnosis for aspergillosis probable.

Galactomannan is a major constituent of aspergillus cell walls that is released during growth of hyphae. A double sandwich enzyme immunoassay (EIA) that detects the galactomannan antigen is available for use on serum and bronchoalveolar lavage (BAL) fluid as an adjunctive test for the diagnosis of aspergillosis with the BAL sample being of much better sensitivity and specificity than serum sample [17].

Empirical antifungal treatment of febrile patients with risk factors for fungal infection was introduced as means to prevent invasive fungal infections in the 1980s after it was noted that many patients with fevers had underlying, otherwise undiagnosed, fungal infections and particularly invasive candidiasis [18].

If an invasive fungal infection is suspected but the diagnosis has not been established, one may consider treating empirically with amphotericin, micafungin, or voriconazole [19]. In case of suspected invasive aspergillosis, the importance of combination therapy has been supported in a recent randomized controlled study by Marr et al. [20] which showed lower mortality rate at six weeks in the combination therapy. On the other hand, disadvantages of combination therapy include greater cost and higher probability of drug to drug interactions and adverse events [21].

Our patient had been started empirically on combination therapy even before the diagnosis made with high suspicion of IFI. Despite that, she did not improve clinically which makes it very important to diagnose and treat this invasive infection aggressively very early.

## 4. Conclusion

We stress the importance of a high degree of clinical suspicion for IFI in transplant patients even many years after transplant. Advocacy for aggressive therapy for such patients is important as they often have poor outcomes despite optimal therapy, which emphasizes the possible benefits of double aspergillosis coverage to decrease mortality as seen in a recent study by Marr et al. [20]. Further randomized controlled studies will support these findings.

## Figures and Tables

**Table 1:** Chronology of infections after heart transplantation [5].

	0-1 month		1–3 months		3–12 months
% of HT patients acquiring an infection	48.6%		27.7%		23.7%

Microbes causing the infection^*∗*^	*Candida Staphylococcus* Nonfoliated *Pseudomonas* Acinetobacter Other fungi CMV	(1.8%) (7.3%) (13.8%) (15.6%) (15.6%) (22%) (26%)	*Aspergillus * *Legionella * Other viruses CMV	(20%) (24.8%) (27%) (28%)	Other bacteria (100%)

Location of infection	Sternotomy Mediastinitis Oropharynx Lung	(7.4%) (12.4%) (16.5%) (64%)	Skin Other Systemic Digestive	(19.9%) (20.4%) (27.6%) (32%)	Genitourinary (100%)

Total % of heart transplant patients acquiring an infection in the first year	48.6%				

^*∗*^Percentage of the number of people in that time period infected with the particular microbe.

## References

[1] Hibberd P. L., Rubin R. H. (1994). Clinical aspects of fungal infection in organ transplant recipients. *Clinical Infectious Diseases*.

[2] Kousha M., Tadi R., Soubani A. O. (2011). Pulmonary aspergillosis: a clinical review. *European Respiratory Review*.

[3] Segal B. H., Walsh T. J. (2006). Current approaches to diagnosis and treatment of invasive aspergillosis. *American Journal of Respiratory and Critical Care Medicine*.

[4] Montoya J. G., Giraldo L. F., Efron B. (2001). Infectious complications among 620 consecutive heart transplant patients at stanford university medical center. *Clinical Infectious Diseases*.

[5] Sánchez-Lázaro I. J., Almenar L., Blanes M. (2010). Timing, etiology, and location of first infection in first year after heart transplantation. *Transplantation Proceedings*.

[6] Pappas P. G., Alexander B. D., Andes D. R. (2010). Invasive fungal infections among organ transplant recipients: results of the transplant-associated infection surveillance network (TRANSNET). *Clinical Infectious Diseases*.

[7] Haddad F., Deuse T., Pham M. (2010). Changing trends in infectious disease in heart transplantation. *Journal of Heart and Lung Transplantation*.

[8] Muñoz P., Cerón I., Valerio M. (2014). Invasive aspergillosis among heart transplant recipients: a 24-year perspective. *Journal of Heart and Lung Transplantation*.

[9] Sambatakou H., Dupont B., Lode H., Denning D. W. (2006). Voriconazole treatment for subacute invasive and chronic pulmonary aspergillosis. *American Journal of Medicine*.

[10] Gavalda J., Len O., San Juan R. (2005). Risk factors for invasive aspergillosis in solid-organ transplant recipients: a case-control study. *Clinical Infectious Diseases*.

[11] Shields R. K., Nguyen M. H., Shullo M. A. (2012). Invasive aspergillosis among heart transplant recipients is rare but causes rapid death due to septic shock and multiple organ dysfunction syndrome. *Scandinavian Journal of Infectious Diseases*.

[12] Montoya J. G., Chaparro S. V., Celis D. (2003). Invasive aspergillosis in the setting of cardiac transplantation. *Clinical Infectious Diseases*.

[13] Playford E. G., Webster A. C., Sorell T. C., Craig J. C. (2004). Antifungal agents for preventing fungal infections in solid organ transplant recipients. *Cochrane Database of Systematic Reviews*.

[14] Singh N. (2000). Antifungal prophylaxis for solid organ transplant recipients: seeking clarity amidst controversy. *Clinical Infectious Diseases*.

[15] Muñoz P., Valerio M., Palomo J. (2013). Targeted antifungal prophylaxis in heart transplant recipients. *Transplantation*.

[16] De Pauw B., Walsh T. J., Donnelly J. P. (2008). Revised definitions of invasive fungal disease from the European organization for research and treatment of cancer/invasive fungal infections cooperative group and the national institute of allergy and infectious diseases mycoses study group (EORTC/MSG) consensus group. *Clinical Infectious Diseases:*.

[17] Meersseman W., Lagrou K., Maertens J. (2008). Galactomannan in bronchoalveolar lavage fluid: a tool for diagnosing aspergillosis in intensive care unit patients. *American Journal of Respiratory and Critical Care Medicine*.

[18] Marr K. A. (2002). Empirical antifungal therapy—new options, new tradeoffs. *The New England Journal of Medicine*.

[19] Oyake T., Kowata S., Murai K. (2015). Comparison of micafungin and voriconazole as empirical antifungal therapies in febrile neutropenic patients with hematological disorders: a randomized controlled trial. *European Journal of Haematology*.

[20] Marr K. A., Schlamm H. T., Herbrecht R. (2015). Combination antifungal therapy for invasive aspergillosis: a randomized trial. *Annals of Internal Medicine*.

[21] Vazquez J. A. (2008). Combination antifungal therapy for mold infections: much ado about nothing?. *Clinical Infectious Diseases*.

